# Four Decades of Inquiry Into the Genetic Bases of Specific Reading Disability

**DOI:** 10.1044/2025_JSLHR-25-00050

**Published:** 2025-10-15

**Authors:** Pavel Dobrynin, Yi Zeng, Marina Norkina, Alina Fedorova, Anna Zhuk, Elena L. Grigorenko

**Affiliations:** a Department of Psychology, University of Houston, TX; b Texas Institute for Measurement, Evaluation and Statistics, University of Houston, TX; c Department of Chemistry, University of Houston, TX; d Center for Cognitive Sciences, Sirius University of Science and Technology, Russia; e International Longevity Alliance, Sceaux, France; f Institute of Applied Computer Science, ITMO University, St. Petersburg, Russia; g Laboratory of Amyloid Biology, St. Petersburg State University, Russia; h Department of Molecular and Human Genetics, Baylor College of Medicine, Houston, TX; i Departments of Pediatrics, Baylor College of Medicine, Houston, TX

## Abstract

**Purpose:**

This study investigated the genetic bases of specific reading disability (SRD) by systematically cataloging candidate genes reported as associated with SRD and reading-related processes over the last 4 decades and analyzing their evolutionary conservation, developmental expression patterns, and functional networks to address significant knowledge gaps in understanding the genetic architecture of reading (dis)ability.

**Method:**

Through a comprehensive literature review, we identified 175 putative SRD (and reading-related processes) candidate genes (hereafter, SRD genes). Using bioinformatic approaches, we analyzed their evolutionary conservation across species, examined their expression patterns in developmental and single-cell transcriptome data sets from the Allen Brain Atlas, and performed functional pathway analyses to identify biological processes associated with these genes.

**Results:**

SRD genes showed remarkable evolutionary conservation, with enrichment in ancient taxonomic groups. Developmental transcriptome analysis revealed two distinct gene clusters with expression differentiation around 24 postconception weeks: early genes associated with brain morphology development and later genes involved in synaptic signaling. Single-cell analysis identified cell-type–specific expression patterns and protein–protein interaction networks with hub genes potentially coordinating reading-related neural pathways.

**Conclusions:**

Our findings challenge the notion of the existence of reading-specific genes, suggesting instead that SRD reflects the disruption of ancient evolutionary neural mechanisms operating within human-specific brain architecture. The identification of developmental expression transitions and functional networks provides insight into how genetic variation might impact reading development and offers potential targets for future clinical approaches to the identification and remediation of reading difficulties.

**Supplemental Material:**

https://doi.org/10.23641/asha.30290446

Specific reading disability (SRD), often, although disputably (Elliott & Grigorenko, 2024), referred to as dyslexia, is a developmental disorder whose features might persist throughout life (*Diagnostic and Statistical Manual of Mental Disorders, Fifth Edition*; American Psychiatric Association, 2013). The public health significance of SRD is difficult to overestimate: It is common, as its prevalence is estimated at 5%–15% (Grigorenko et al., 2020); its remediation consumes substantial resources, as individuals with reading difficulties constitute 32% of those supported by the IDEA (Individuals with Disabilities Education Act; Pendharkar, 2023); and it is comorbid with negative life outcomes (Cummings & Luengo-Prado, 2023). SRD is phenomenologically and etiologically complex; its emergence reflects the convergence of environmental and genetic risk factors (Elliott & Grigorenko, 2024). The search for the latter formally began with the 1983 publication in *Science* (Smith et al., 1983), which engaged both diagnostic categories and continuously distributed traits characterizing reading and related processes.

The overwhelming majority of molecular genetic studies have propelled the general assumption that SRD arises, at least in part, due to some structural variation in the genome. Since 1983, a variety of designs and sampling, molecular, bioinformatical, and statistical methods have been utilized in the search for this variation. In a systematic review of the literature, we identified peer-reviewed publications—regardless of the writing system studied—that reported the results of such analyses. From these, we extracted the names of genes labeled by the authors as “candidate” or “putative candidate” genes for SRD. The features of the resulting 175 genes (hereafter referred to as SRD genes; Supplemental Material S1: SRD Genes) were analyzed to address several knowledge gaps in our understanding of the genetic bases of reading ability and disability, hereafter referred to as reading (dis)ability.

These knowledge gaps are as follows. First, there is no comprehensive catalog of candidate genes identified across diverse methodologies and languages, limiting our understanding of the genetic landscape underlying reading (dis)ability. Second, while individual genes have been characterized in detail, their collective evolutionary conservation and novelty patterns remain unexplored. Third, their developmental expression profiles across brain regions and cell types have neither been systematically analyzed as functional groups nor have the biological pathways connecting them to reading (dis)ability been well described. To address these gaps, we pursued four objectives: (a) compiling and analyzing the SRD candidate genes reported over 4 decades; (b) examining their evolutionary conservation and novelty; (c) characterizing their developmental and cell-type–specific expression in the human brain; and (d) identifying functional pathways linking these genes to reading development. By integrating evolutionary and transcriptomic analyses, we aimed to provide novel insights into how genetic factors contribute to reading (dis)ability, potentially informing future clinical approaches for identification and remediation.

## Materials and Method

### Initial Literature Review

To compile a comprehensive list of the SRD genes, we conducted a systematic review focusing on English-language publications from 1983 to 2023, regardless of the writing systems and languages studied. Our search criteria included various types of research methods, such as linkage and association studies, as well as studies on dyslexia and reading disorders, however specified. We also considered studies within linkage regions associated with reading-related phenotypes, as well as those examining candidate genes previously associated with reading (dis)ability samples. Case studies, group studies, and research on common endophenotypes such as single-word reading, pseudoword decoding, phonological processing, and vocabulary, among others, were included. We excluded studies that focused on genes associated with other disorders, such as cancer, even if they included the reading-related phenotype. A detailed description of the criteria for each search step is available in Supplemental Material S2: Literature Review Methodology.

### Gene Expression Data Sets

*Developmental transcriptome data*. We analyzed preprocessed transcriptomic data from the BrainSpan Atlas of the Developing Human Brain, which is part of the Allen Brain Atlas resources (https://www.brainspan.org/static/download.html). These data were originally generated through RNA (ribonucleic acid) sequencing and exon microarray analyses and cover multiple cortical and subcortical brain regions across the full spectrum of human neurodevelopment.

*Single-cell transcriptome data*. We analyzed preprocessed single-nucleus transcriptomic data from the Allen Brain Atlas, derived from 76,533 nuclei extracted from two postmortem human primary motor cortex (M1C or M1) specimens. These data describe 127 distinct transcriptomic cell types (https://portal.brain-map.org/atlases-and-data/rnaseq/human-m1-10x).

### Gene Expression Analysis of Developmental Transcriptome Data

We obtained gene expression data for various brain structures at different ages from the Allen Brain Atlas (Sunkin et al., 2012) to explore the expression profiles of the SRD genes. Data filtering and manipulation were performed using the Phantasus web application (Kleverov et al., 2024; Zenkova et al., 2020). Differential expression analysis was conducted with Limma (Linear Models for Microarray and RNA-Seq Data; Ritchie et al., 2015). From the 26 brain regions available in the Atlas, we selected 16 (see Supplemental Material S3: Brain Regions in Transcriptome Analysis) that had the most time points (*n* > 10). Time points were uniformly converted into postconception weeks (pcw) format for consistency. The filtered gene expression matrix was then imported into Phantasus, where we utilized built-in tools for subsequent processing. Initially, quantile normalization was applied to ensure uniformity across expression data. Subsequently, the data were filtered to focus only on SRD genes. To visualize the expression patterns across all regions, a final heatmap was generated using hierarchical clustering algorithms with one minus Pearson correlation as a distance metric. For differential gene expression analysis, we employed Limma, categorizing time points into prenatal and postnatal groups. Default Limma parameters were utilized for consistency.

### Gene Expression Analysis of Single-Cell Transcriptome Data

We used single-cell gene expression data from the Allen Brain Atlas. Data processing and visualization were performed with Python in the Google Colab environment. Moreover, the Ward method was used for the hierarchical clustering of genes and cell types. The Ward method is a hierarchical clustering approach that minimizes the total within-cluster variance by merging clusters that result in the smallest increase in the sum of squared distances between each data point and its cluster centroid. This method is particularly effective for gene expression data as it tends to create coherent clusters of similar expression patterns. Gene IDs from these clusters were mapped to corresponding proteins using the STRING database (Szklarczyk et al., 2021) to retrieve high-confidence protein–protein interaction (PPI) data for Homo sapiens. Subsequently, we performed functional enrichment analysis on the gene clusters to interpret their biological significance and identify enriched pathways and processes.

### Signals of Selection Analysis

Using a list of SRD genes obtained through our systematic review, we extracted the corresponding single-copy orthologous groups, CDS (Coding DNA Sequence) sequences, and protein sequences from the NCBI (National Center for Biotechnology Information) GenBank database (Wheeler et al., 2007). The analysis focused on the following species: *Homo sapiens*, *Pan troglodytes*, *Pan paniscus*, *Gorilla gorilla*, and *Pongo abelii*. Out of the 175 SRD genes, only 92 were annotated in all five studied genomes and were used for analysis; 83 SRD genes were excluded. To investigate evolutionary selection acting on candidate genes, we applied a combination of complementary bioinformatics tools that assess selection pressure at the codon level. Protein alignments were performed using Clustal Omega (Sievers & Higgins, 2018) with default parameters for initial multiple sequence alignments of orthologous protein sequences. We employed PAL2NAL (Suyama et al., 2006) to overlay protein alignments onto CDS sequences, generating codon-aware nucleotide alignments while preserving reading frame integrity, using the *-nogap* and *-output paml* format options. These codon alignments served as input for selection analyses. Alignment quality is crucial for accurately detecting positive selection, as alignment errors can lead to unacceptably high false positives when using the branch-site model (Yang & dos Reis, 2010). To ensure high-quality alignments, we applied an additional filtering step using SWAMP (Sliding Window Alignment Masker for PAML; Harrison et al., 2014); we excluded genes with sequence properties known to often result in false positives, such as a high proportion of low complexity or disordered regions, ubiquitous domains, repeats, and others. The species tree was extracted from the mammal Zoonomia tree, which is based on alignments of 242 genomes (Zoonomia Consortium, 2020).

To identify genes that have undergone selection, we employed PAML (Phylogenetic Analysis by Maximum Likelihood), a maximum-likelihood method for analyzing molecular evolution (Yang, 2007). We tested several models: the branch model with multiple ω ratios for the human branch (testing whether nonsynonymous/synonymous substitution rate ratios differ on a specified lineage), the branch-site test comparing Model A0 (allowing sites to evolve neutrally and under purifying selection) with Model A (allowing sites under positive selection), and site tests comparing Models M0, M1, M2, M7, and M8. Fixed ω models were also tested to improve interpretability and model stability in cases of low substitution counts.

Applying PAML models revealed no significant evidence for positively selected sites in the human lineage when compared to other representatives of the Hominidae family. For negative selection testing, we used the FEL (fixed-effects likelihood) model (Pond & Frost, 2005) from the HyPhy package (Kosakovsky Pond et al., 2020) to identify codon sites evolving under pervasive selection across the phylogeny, which confirmed a high degree of conservation at the amino acid level, indicating that negative selection rather than positive might be a predominant force shaping these genes. To explore the evolutionary dynamics from a different perspective, we employed the RELAX model (Wertheim et al., 2015), which tests whether selection has been systematically relaxed or intensified on a specified lineage. RELAX is particularly sensitive to shifts in selective constraint, even in the absence of strong positive selection at specific sites.

We selected these complementary tools for their broad acceptance in the field and robustness to different evolutionary scenarios. While PAML offers a powerful parametric model fitting for branch-level selection, FEL provides site-specific resolution, and RELAX detects more subtle global shifts in selection pressure—all of which align with our goal of characterizing evolutionary forces acting on the genes of interest in a lineage-specific manner.

### Evolutionary Novelty Analysis

We used our list of 175 SRD genes to assess their evolutionary novelty by searching orthologs for each SRD gene in the HomoloGene Database, which is also part of the NCBI website (Wheeler et al., 2007). HomoloGene is a database of both curated and computed gene orthologs. We used the approach previously developed by our group to analyze novel genes in the *H. sapiens* lineage (Dobrynin et al., 2013). As an input, the program uses gene name and/or taxon name, and the output is clusters of orthologs. For this study, we queried the HomoloGene database for all protein-coding genes in the *H. sapiens* genome. The taxonomic levels in HomoloGene naturally organized ortholog groups of *H. sapiens* genes into 11 distinct evolutionary groups, representing major transitions in evolutionary history: Eukaryota, Opisthokonta, Bilateria, Euteleostomi, Tetrapoda, Amniota, Boreoeutheria, Catarnini, Euarchontoglires, Homininae, and *H. sapiens*. This classification allowed us to determine whether SRD genes are enriched in evolutionary novel genes (those appearing recently in human evolution) or in more ancient genes. The distribution pattern of SRD genes across these 11 evolutionary groups was then compared with the distribution of all human genes to identify any significant differences that might reveal the evolutionary basis of reading ability. The rationale for this analysis was to test whether an evolutionary novel trait (reading) corresponds with evolutionary novel genes. The analysis was performed using a custom Python script (code available in Supplemental Material S10) and raw data from the HomoloGene database (available in Supplemental Material S11).

## Functional Analysis of SRD Genes

### Gene Ontology and Pathway Enrichment Analysis

To understand the biological functions of SRD genes, we performed gene set enrichment analysis using the ShinyGO platform (Ge & Jung, 2018), identifying overrepresented Gene Ontology (GO; The Gene Ontology Consortium, 2023) terms related to biological processes and cellular components. Enrichment analysis was conducted separately for the differentially expressed gene clusters identified in our developmental transcriptome analysis. For pathway analysis, we utilized the Kyoto Encyclopedia of Genes and Genomes (KEGG; Kanehisa et al., 2023) database to identify metabolic and signaling pathways associated with the SRD genes. Additionally, we employed Reactome (Gillespie et al., 2022), a comprehensive database of biochemical reactions and pathways, to further characterize the functional implications of these genes. For all enrichment analyses, terms with a false discovery rate (FDR) < 0.05 were considered significant.

### PPI Network Analysis

To explore functional relationships between the SRD genes in a single-cell transcriptome data set, we constructed PPI networks using the STRING database. For each constructed network, STRING calculated standard network parameters, including average node degree, expected number of edges, and clustering coefficient. Statistical significance of the networks was determined by comparing the observed number of edges against the expected number based on random gene sets of equivalent size.

## Results

### Gene Expression Analysis of Developmental Transcriptome Data

**Figure 1.**
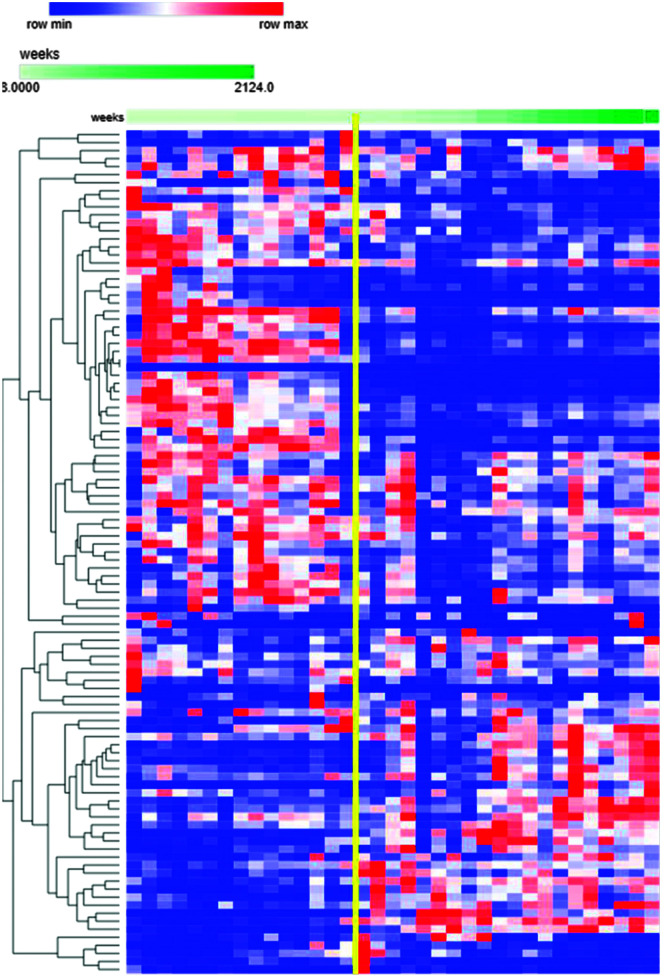
Heatmap depicting specific reading disability transcription levels in the ventrolateral prefrontal cortex. Columns represent distinct samples, and rows represent individual genes. Time is measured in weeks after conception, ranging from 3 postconception weeks to 40 years of age. The vertical yellow line denotes 24 weeks.

All examined regions displayed similar patterns of gene expression (see Figure 1), marked by a distinct transition in gene expression levels around 24 weeks. The expression levels of the SRD genes were compared between fetal and all other life periods (i.e., all samples acquired at different developmental stages), revealing 126 significantly (adjusted *p* value < .05) differentially expressed genes (see Supplemental Material S4: Differential Gene Expression Analysis). Among these, 68 genes were upregulated during fetal development. These genes play pivotal roles in critical processes associated with fetal brain development, including cell morphogenesis (GO:0048667, FDR = 2.00E-10), axonogenesis (GO:0007409, FDR = 3.09E-09), and neurogenesis (GO:0022008, FDR = 8.23E-08), and the axon guidance (GO:0007411, FDR = 2.95E-07), essential for establishing neural networks (see Supplemental Material S5: Prenatal Gene Expression Enrichment). In total, gene set enrichment analysis revealed 199 (FDR *<* 0.05) GO groups (GO: Biological processes) enriched during early development.

The second cluster, consisting of 58 genes, exhibited elevated expression levels after 24 pcw and postnatally. Among those genes, there were 196 significantly (FDR *<* 0.05) enriched GO groups (GO: Biological processes). These genes are integral to the functioning of the nervous system and the development of cognition (see Supplemental Material S6: Postnatal Gene Expression Enrichment). They are involved in signaling pathways related to following GO terms: learning or memory (GO:0007611, FDR = 1.00E-3), cell projection organization (GO:0030030, FDR = 1.00E-3), modulation of chemical synaptic transmission (GO:0050804, FDR = 5.00E-3), regulation of calcium ion transport (GO:1901019, FDR = 5.00E-3), trans-synaptic signaling (GO:0099537, FDR = 1.00E-2) and neurotransmitter secretion (GO:0007269, FDR = 1.00E-2). These findings directly address our third research objective by characterizing the developmental expression profiles of the SRD genes in the human brain, revealing a critical transition point at 24 pcw that distinguishes genes involved in early brain morphology from those supporting later synaptic function.

### Gene Expression Analysis of Single-Cell Transcriptome Data

**Figure 2.**
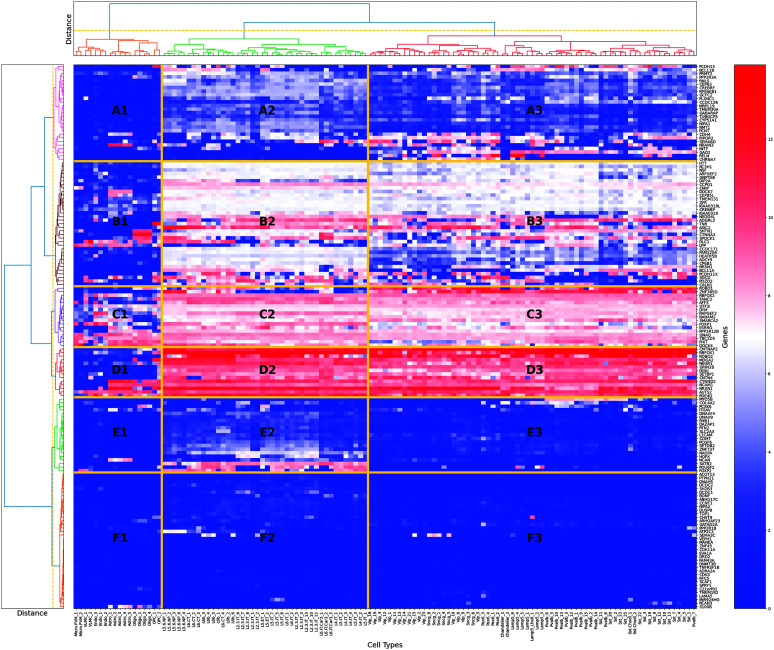
Clusters of specific reading disability gene expression based on single-cell brain data from 128 cells.

Additionally, we explored the SRD gene expression profile in a single-cell data set from the Allen Brain Atlas. Using Ward's hierarchical clustering method, we demarcated six distinct (see Figure 2, gene clusters from A to F) gene expression patterns across various brain cell types. The brain cell types (see Table 1) were divided into three primary cell clusters: (a) Non-Neuronal Cells: Including Astrocytes (Astro), Oligodendrocyte Precursor Cells (OPC), Oligodendrocytes (Oligo), Endothelial Cells (Endo), Vascular Leptomeningeal Cells (VLMC), and Microglia/Perivascular Macrophages (Micro.PVM); (b) Cortical Excitatory Neurons: Comprised of Layer 2/3 Intratelencephalic neurons (L2.3.IT), Layer 6 Intratelencephalic neurons (L6.IT), Layer 4 Intratelencephalic neurons (L4.IT), Layer 5 Intratelencephalic neurons (L5.IT), Layer 6 Corticothalamic neurons (L6.CT), Layer 6b neurons (L6b), Layer 6 Intratelencephalic Car3 neurons (L6.IT.Car3), and Layer 5/6 Near-Projecting neurons (L5.6.NP); (c) Cortical Inhibitory Neurons and Pax6-Associated cells: Including Lamp5-Expressing interneurons (Lamp5), Pax6-Expressing cells (Pax6), Sncg-Expressing interneurons (Sncg), Vasoactive intestinal peptide interneurons, Somatostatin-Chodl interneurons (Sst.Chodl), Somatostatin interneurons (Sst), Parvalbumin interneurons (Pvalb), and Chandelier cells (Chandelier). Cell type classifications were defined according to the Allen Brain Atlas single-cell database (https://portal.brain-map.org/atlases-and-data/rnaseq/human-m1-10x). A cluster of Non-Neuronal Cells (Cell Cluster 1) generally demonstrated lower expression levels across most gene clusters, with distinct patches of high expression in gene clusters C and D and low expression in cluster B. Cortical Excitatory Neurons cluster (Cell Cluster 2) exhibited high expression in gene clusters B, C, and D and varied expression patterns in cluster A and low expression in cluster F. Cortical Inhibitory Neurons and Pax6-Associated cells (Cell Cluster 3), similar to Cell Cluster 2, displayed high expression in gene clusters B, C, and D. Across all cell clusters, gene cluster F showed consistently low expression, while cluster C displayed consistently high expression levels. Cell Cluster 1 was the most divergent across the three cell clusters, and Cell Clusters 2 and 3 demonstrated high levels of similarity in gene expression patterns, with the exception of gene cluster E, which was more active in Cell Cluster 2.

**Table 1.** Summary of differential gene expression across brain cell-type clusters.

Gene cluster	No. of genes	Differentially expressed	Upregulated	Downregulated
A	23	18	9	9
B	30	28	17	11
C	15	13	7	6
D	13	9	2	7
E	19	16	7	9
F	33	21	10	11

We also annotated the SRD genes using differential gene expression data from a developmental transcriptome data set from the Allen Brain Atlas. Each SRD gene from the developmental transcriptome data set was annotated with cluster ID derived from single-cell data (see the Single-cell gene cluster column in Supplemental Material S4: Differential Gene Expression Analysis). The distinct expression patterns across gene clusters highlighted the molecular diversity of the brain cell types.

**Figure 3.**
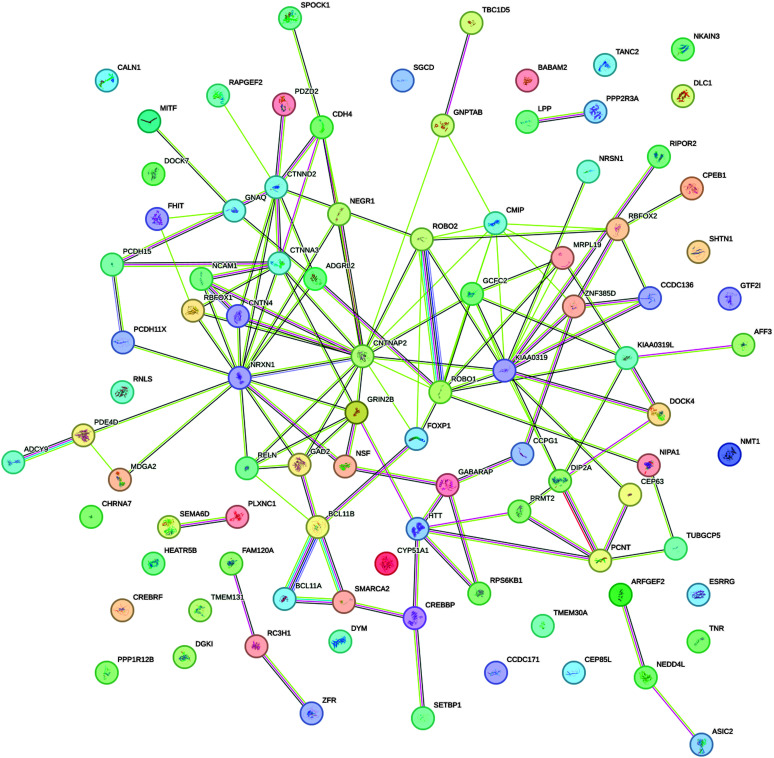
Protein–protein interaction network for 92 specific reading disability genes from clusters A, B, C, and D. The edges (lines connecting proteins) indicate predicted functional associations. The network view is evidence based, meaning each edge may display up to eight distinct colored lines, each representing a different type of evidence supporting the prediction. A higher number of edges between two nodes represents higher confidence of interaction between them. For details on the color codes, refer to the STRING documentation (https://string-db.org/).

**Figure 4.**
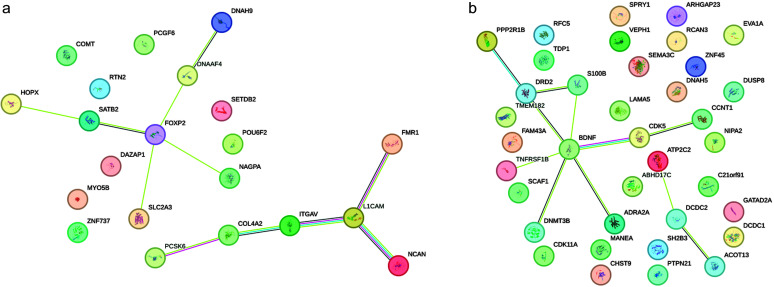
Protein–protein interaction networks for gene clusters E (a) and F (b). The network view is evidence based, meaning each edge may display up to eight distinct colored lines, each representing a different type of evidence supporting the prediction. A higher number of edges between two nodes represents higher confidence of interaction between them. For details on the color codes, refer to the STRING documentation (https://string-db.org/).

The largest cluster, F, included 38 SRD genes, with almost no expression in the cell types explored here. More than 60% of genes in cluster F were differentially expressed developmentally, suggesting that most of their activity is characteristic of different brain cell types not present in the single-cell sample group analyzed here. Nevertheless, 40% of the cluster F genes were not differentially expressed, highlighting the elusive nature of gene activity in this cluster, as the currently available information suggests. The sparse expression pattern in Cell Cluster 1 of Non-Neuronal Cells indicated the very limited activity of SRD genes in glial functions such as maintaining brain homeostasis, supporting neuronal function, and mediating immune responses in the CNS. The high expression in clusters B, C, and D for both excitatory and inhibitory neurons suggested that these clusters contained genes fundamental to neuronal function, such as those involved in neurotransmission or synaptic plasticity. Only B and D clusters demonstrated preferential time-dependent activity; D was more active in prenatal development, while B was in postnatal development. Based on identified similarities in gene activity for clusters A, B, C, and D, we combined them into a single mega-cluster of 92 genes for network and gene set enrichment analysis in STRING. The PPI network for the SRD genes from clusters A, B, C, and D (see Figure 3) had a significantly higher (129) than expected (34) number of edges (*p* = 1.0E-16). These 92 genes were part of the 175 SRD genes that we initially identified through our systematic literature review (as described in the Materials and Methods section and listed in Supplemental Material S1) and were specifically selected based on their expression patterns in the single-cell analysis showing similar activity levels in neuronal cell types. On average, every gene was connected to three other genes in the network (average node degree = 2.8), with some of the genes (*NRXN1*, *CTNNA3*, *CTNNAP2*, *KIAA0319*, *CMIP*, *ROBO1*, *GRIN2B*) demonstrating a high number of edges and hub formation. The constructed network was enriched in proteins related to neuron projection (GO:0043005, FDR = 4.41E-12). We also explored the PPI network for gene clusters E and F. Cluster E demonstrated some levels of expression almost exclusively in excitatory neurons, and networks drawn from its 21 genes (see Figure 4a) had two connected components with *FOXP2* and *L1CAM* in the role of a central hub for each. The network (see Figure 4b) based on gene cluster F (37 genes) had the least average node degree and clustering coefficient, stressing the loose structure of this network. There were two connected components, with three and nine genes, respectively, and the *BDNF* gene was a hub for a larger group.

This specific cell-type (see Figure 2) analysis fulfills our third research objective by identifying distinct expression patterns of SRD genes across neuronal and non-neuronal cell populations. Thus, the identification of distinct gene units with varying expression patterns across neuronal and non-neuronal cell types, particularly the highly expressed genes in Clusters B, C, and D in both excitatory and inhibitory neurons, highlights potential key players in neuronal function related to reading. Furthermore, the PPI network analysis revealed significant interconnectivity among SRD genes, with hub genes like *NRXN1*, *CTNNA3*, and *ROBO1* emerging as critical nodes, suggesting their central role in coordinating molecular processes underlying reading-related (and perhaps language-related) brain pathways.

### Signals of Selection

We explored signals of positive selection among the SRD genes. The analysis focused on the following species: *Homo sapiens*, *Pan troglodytes*, *Pan paniscus*, *Gorilla gorilla*, and *Pongo abelii*. No significant evidence for positively selected sites in the human lineage when compared to other representatives of the *Hominidae* family was obtained (see Supplemental Material S7: Evolutionary Selection Analysis). Intriguingly, our analysis revealed evidence of intensified selection on three genes, suggesting a nuanced evolutionary scenario that is not captured by traditional positive selection models provided by PAML and its derived tools. Specifically, *POU6F2* and *MDGA2*, genes associated with neural development and brain function, along with *SH2B3*, a gene implicated in immune response and hematopoiesis, exhibited evidence of intensified selection. This suggests a potential evolutionary emphasis on brain development and immune response adaptations in humans. The identification of intensified selection, despite the absence of evidence for positive selection, highlights the complexity of evolutionary processes and underscores the need for a multiplicity of analytical lenses to understand them fully. These evolutionary findings address our second research objective by demonstrating that SRD genes are predominantly under purifying selection, with limited evidence of positive selection in the human lineage, suggesting functional constraint rather than recent adaptation.

### Evolutionary Novelty

**Figure 5.**
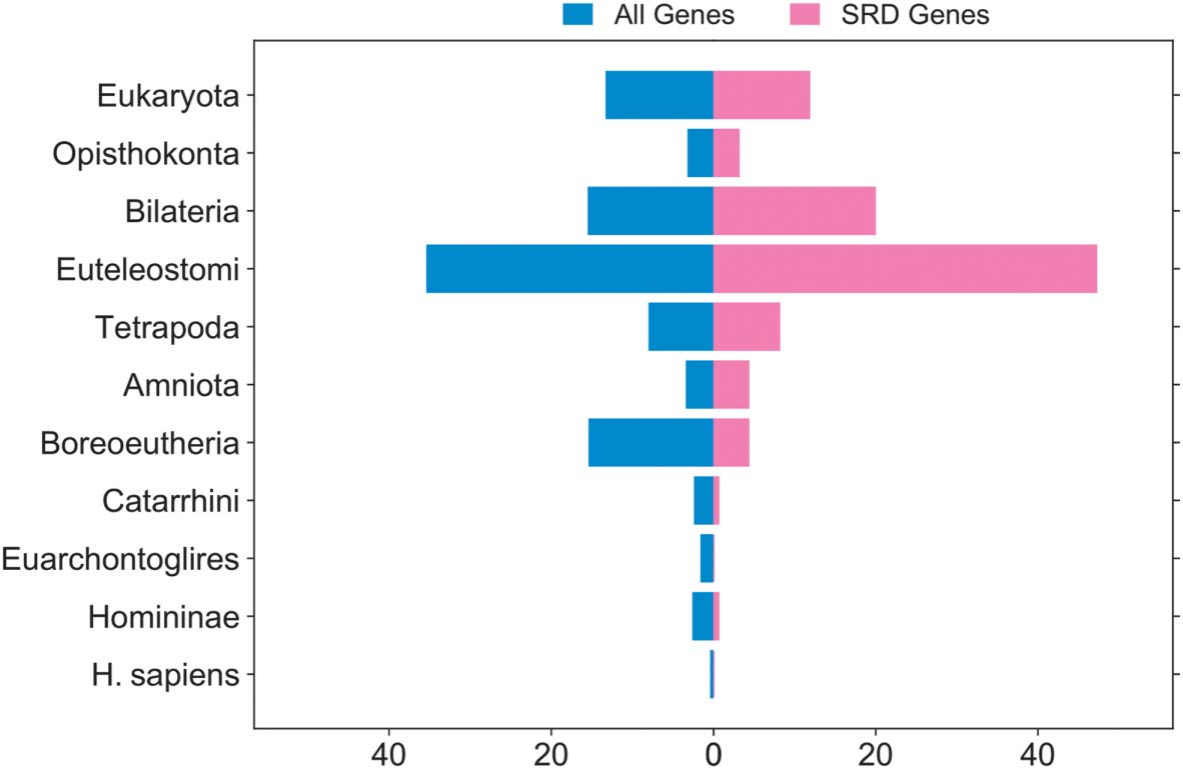
The distribution of the specific reading disability (SRD) genes in homologous sequence groups. SRD-related genes are notably enriched in groups emerging at the Bilateria and Euteleostomi levels.

To evaluate the evolutionary novelty of SRD genes, we analyzed homologous sequences from the NCBI HomoloGene database. Our results (see Figure 5) reveal that the SRD genes are rarely evolutionarily recent; instead, most predate the majority of novel human genes, with pronounced enrichment in ancient lineages—particularly Bilateria (organisms with bilateral symmetry and cephalized nervous systems) and Euteleostomi (bony vertebrates, including fish and tetrapods). This pattern highlights their deep conservation and suggests that their initial roles in fundamental processes, such as neural organization and brain development, were later co-opted for human-specific traits, including reading. This analysis completes our examination of evolutionary patterns (our second objective) by revealing that SRD genes are predominantly ancient rather than an evolutionary novel in origin, challenging assumptions about reading-specific genetic adaptations.

### Functional Analysis of SRD Genes

**Figure 6.**
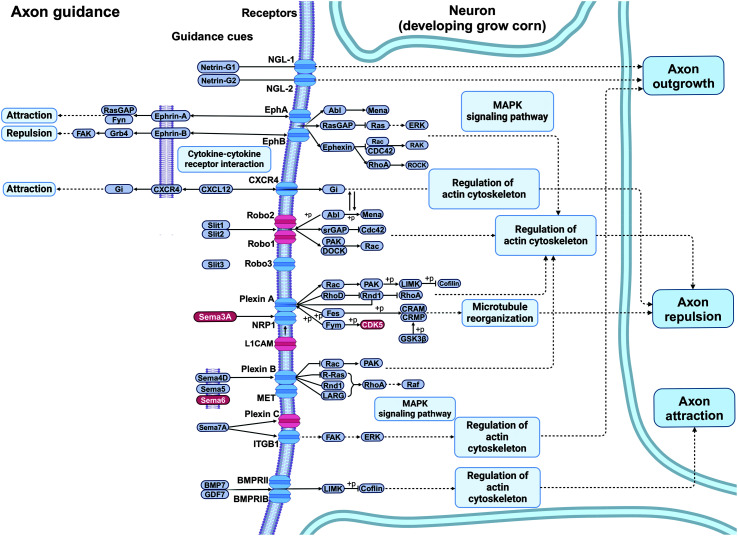
Simplified version of the KEGG (Kyoto Encyclopedia of Genes and Genomes) axon guidance pathway with a focus on repulsion. Genes from the specific reading disability list are marked in red. MAPK = mitogen-activated protein kinase.

**Figure 7.**
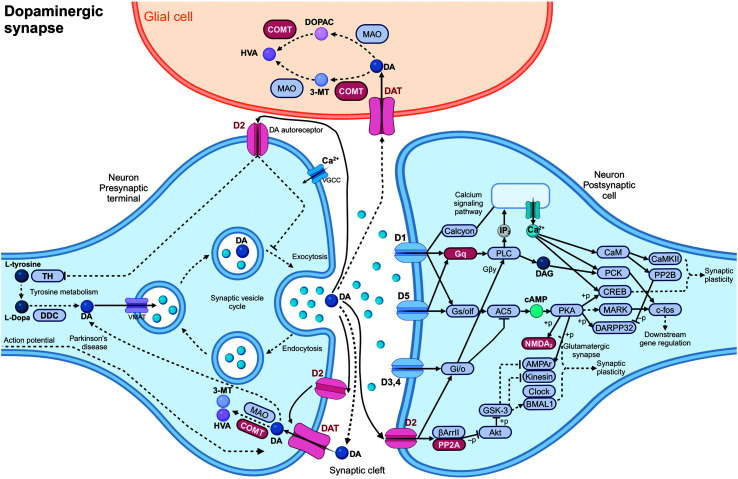
Simplified version of the KEGG (Kyoto Encyclopedia of Genes and Genomes) dopaminergic synapse pathway. Genes from the specific reading disability list are marked in red.

Gene set enrichment analysis revealed enriched GO terms related to biological processes and cellular components. These analyses demonstrated pronounced enrichments in categories associated with neurogenesis, development, and axon and dendrite formation. Enrichment in neuronal and developmental categories is expected, but specific categories in the top list still provide information about the major processes involved. There was a large overlap in gene content for the most significant GO categories. The analysis of the KEGG database, integrating information on genes, proteins, and pathways, and providing a comprehensive resource for the analysis of biological systems and their functional implications, demonstrated significant enrichment in four KEGG metabolic pathways: (a) axon guidance, (b) dopaminergic signaling, (c) extracellular Matrix (ECM)–receptor interaction, and (d) phosphatidylinositol 3-kinase-protein kinase B (PI3K-Akt) signaling (see Supplemental Material S8: KEGG Pathway Enrichment and Figures 6 and 7). ECM–receptor interaction and PI3K-Akt signaling capture general cellular interactions and form signaling pathways closely associated with each other; they are not presented in detail, given the nonspecificity of their function. The other two pathways directly relate to nervous system development and are therefore illustrated here. For the axon guidance pathway (see Figure 6), eight genes were flagged: seven of them (*SEMA3F*, *SEMA3C*, *SEMA6D*, *CDK5*, *ROBO1*, *ROBO2*, *L1CAM*) substantiate axon repulsion. In the dopaminergic synapse pathway (see Figure 7), the gene list comprised the following seven genes: *PPP2R3A* (PP2A), *COMT*, *PPP2R1B* (PP2A), *DRD2* (D2), *GNAQ* (Gq), *GRIN2B* (NMDAr), and *SLC6A3* (DAT). Notably, *DRD2* and *SLC6A3* are responsible for dopamine receptor and transporter functions, which are essential for synaptic transmission. The other genes influence various aspects of dopamine metabolism (*COMT*) or signal transduction (*PPP2R3A*, *PPP2R1B*, *GNAQ*, *GRIN2B*).

Using Reactome, a database of signaling and metabolic molecules organized into biological pathways and processes, we identified six enriched terms (see Supplemental Material S9: Reactome Pathway Analysis). Most of these terms correspond to similar groups found in our other analyses. One notable exception is “Transcriptional Regulation by MECP2.” Methyl-CpG binding protein 2 (*MECP2*) is a protein that binds to methylated DNA, playing a crucial role in gene expression regulation and neuronal development. Mutations in this gene are strongly associated with Rett syndrome, a severe neurodevelopmental disorder that predominantly affects females and manifests in a broad spectrum of neurological and developmental impairments.

Using gene set enrichment analysis and data from multiple databases, we registered the presence of the SRD genes in the pathways regulating neurogenesis, axon guidance, dopamine signaling, and transcriptional regulation by *MECP2*. These findings shed light on the roles of the SRD genes in critical pathways related to neurodevelopment and neuronal function. Recent comparisons of the currently available genomes from multiple species have enabled the discovery of noncoding sequences with higher substitution rates in the *H. sapiens* lineage. Additionally, thousands of human-accelerated regions (HARs) have been identified, many of which play crucial roles in brain development and cognitive functions. Using the list of known HARs, we additionally checked their presence in proximity to the SRD genes. Almost 10% of the SRD genes were colocated with HARs, underscoring the role of the evolution of regulatory genome sequences in human brain evolution (Bi et al., 2023). These functional pathway findings fulfill our fourth research objective by identifying biological networks potentially linking the SRD genes to reading development, particularly highlighting axon guidance, dopaminergic signaling, and *MECP2* regulation as critical pathways.

## Discussion

Our investigation of 175 SRD candidate genes identified through a systematic literature review has addressed the knowledge gaps outlined in our objectives. First, by cataloging genes reported across 4 decades of research using diverse methodologies, we have created a foundation for understanding the genetic landscape of reading (dis)ability. Second, our evolutionary analyses revealed that the SRD genes exhibit remarkable conservation and relatively ancient origins, with enrichment at the Bilateria and Euteleostomi levels, challenging notions about reading-specific genetic adaptations. Third, our developmental expression analyses identified a critical transition point around 24 pcw, with two distinct functional clusters emerging—one associated with early brain morphological development and another with later synaptic signaling processes. Finally, our cell-type–specific analyses revealed expression patterns across neuronal and non-neuronal cell populations, with several hub genes potentially coordinating reading-related neural pathways.

These findings collectively provide valuable insights into the genetic bases of reading (dis)ability while raising important questions about the nature of SRD as a specific neurodevelopmental condition rather than an outcome of brain development processes.

### Developmental Expression Patterns of SRD Genes

In examining the developmental expression patterns of the SRD genes, our transcriptomic analyses revealed important temporal distinctions in gene activity. In terms of transcriptional regulation, the human brain is not significantly different from that of chimpanzees. However, it features a more active and intricate transcriptional regulation network (Suntsova & Buzdin, 2020). Remarkably, we observed that the majority (75%) of the SRD genes are differentially expressed in the brain during human development. The two co-expressed gene clusters identified in our developmental transcriptome analysis (the 68 genes upregulated before 24 pcw associated with brain morphology development and the 58 genes upregulated after 24 pcw involved in synaptic signaling) demonstrated some levels of specialization. With the gene set enrichment analysis, we demonstrate the presence of two functional clusters: (a) the first cluster of genes mostly active before 24 pcw are enriched in GO terms related to the development of the brain morphology, and (b) genes in the second cluster are associated with synaptic contacts and signal processing. Activation of the genes from the second cluster only after Week 24 could mean that something significantly changes in our capacity to process information at that developmental juncture. Identification of different co-expression gene clusters and their respective roles could improve our understanding of SRD emergence and its subtypes. The previously reported demarcation period in gene expression (Li et al., 2020) is consistent with our results, but there was no significant overlap between differentially expressed genes in both data sets. These discrepancies could be attributed to the differences in filtering for the differential expression analyses. Expression profiles of all studied brain regions were consistent with each other; this suggests the ubiquitous nature of structures and processes regulated by the SRD genes.

### Cell-Type–Specific Expression Profiles

The expression patterns of the SRD genes across different brain cell types, as revealed by our single-cell analysis, provide further insight into the neural substrates of reading (dis)ability. The high expression of gene clusters B, C, and D in both excitatory and inhibitory neurons suggests that these genes play crucial roles in general neuronal function and plasticity rather than being specific to a particular neural circuit. This broad involvement across neuron types aligns with the idea that reading, as a relatively recent cultural invention, co-opts and repurposes existing neural structures and functions. The PPI networks we constructed, especially those centered around hub genes like *NRXN1*, *CTNNA3*, and *ROBO1*, highlight the interconnected nature of these genes' functions. These hubs may serve as nodes in coordinating the neural processes underlying reading. Interestingly, the large gene group F, which contains genes with almost no expression in the explored cell types but showing differential expression in developmental data sets, suggests the existence of additional cellular contexts relevant to reading development that are not represented in current single-cell RNA data.

### Evolutionary Conservation of SRD Genes

Despite the rapid evolution of cognitive functions observed in primates (Bouret et al., 2024; Rickelton et al., 2024), the SRD genes are constrained in their sequence evolution and demonstrate a relatively ancient origin. Recent phylogenomic analyses of 50 primate species have revealed a remarkable degree of conservation in genes specifically expressed in the brain (Shao et al., 2023). Furthermore, the conservation of protein sequences is notable, particularly for tissue-specific and brain-specific genes (Liao & Zhang, 2006; Wang et al., 2007). These evolutionary constraints are most likely attributed to the high complexity of interaction networks for those genes.

Our analysis of the evolutionary novelty of the SRD genes revealed significant enrichment in ancient taxonomic groups (Bilateria and Euteleostomi) rather than in recent evolutionary innovations. This pattern suggests that reading, despite being a human-specific cognitive skill that emerged culturally only a few thousand years ago, relies on genetic pathways that evolved hundreds of millions of years earlier. The emergence of bilateral symmetry (Bilateria) established distinct left and right sides in organisms, while the rise of Euteleostomi marked the appearance of bony vertebrates with more complex nervous systems—both representing critical evolutionary milestones that laid the foundations for neural structures later co-opted for reading.

This finding aligns with the “neuronal recycling hypothesis,” which proposes that cultural inventions such as reading repurpose evolutionarily older brain circuits for new functions (Dehaene, 2009). The predominance of ancient genes in our SRD gene set supports this view, indicating that reading disabilities likely reflect disruptions in fundamental neural processes that evolved for other purposes and were later adapted for reading acquisition. This evolutionary perspective also helps explain why reading disabilities often co-occur with other neurodevelopmental disorders—they may share disruptions in common, ancient genetic pathways serving multiple functions in brain development.

We observed that only one aspect of recent human evolution appears to be associated with the SRD genes, noncoding HARs. Ten percent of the SRD genes are associated with at least one HAR, emphasizing the role of these regulatory elements in the capacity of *H. sapiens* to acquire new skills, in this case, the skill of reading.

This finding suggests that while the core genetic machinery supporting reading is ancient, human-specific modifications to gene regulation may have been critical in enabling the neural plasticity required for reading acquisition. Thus, the evolutionary profile of the SRD genes provides an important context for understanding reading (dis)ability. The ubiquitous nature of these genes across different brain regions, coupled with their ancient evolutionary origin, suggests that our ability to read is based on preexisting neural structures and functions that evolved long before reading emerged as a cultural skill. This may explain why no nonhuman animals have demonstrated the capacity to acquire reading skills despite sharing many of the same genes—the specific configuration and expanded size of the human brain may enable these ancient genetic pathways to support novel, complex, cognitive functions like reading.

### Functional Significance and Implications

The ubiquitous nature of the SRD genes is concordant with their early evolutionary origin and protein sequence conservativeness. In previous studies, numerous human genes related to brain development were identified as positively selected (Shao et al., 2023), specifically genes that could be associated with increased brain size. Notably, no SRD genes displayed properties of recent origin and/or positive selection. Together with the nearly identical behavior of the SRD genes in different brain regions, this observation provides additional support to the conclusion that our ability to read is based on preexisting brain structures and functions that evolved long before we produced reading skills as a species. The fact that reading is a human-exclusive ability, yet the SRD genes are ancient and conserved across species, suggests an intriguing paradox. Our findings indicate that while the genetic components supporting reading (dis)ability evolved long before reading itself, these genetic elements alone are insufficient for reading acquisition. What makes human reading possible appears to be not specialized “reading genes” but rather how these ancient genetic mechanisms interact within the context of the increased size and complexity of the human brain.

This suggests a two-level model: (a) ancient, conserved genes that support general neural development and function, which are shared across species, and (b) human-specific brain architectural properties—potentially influenced by positively selected genes related to brain size and complexity that are distinct from the SRD genes. Reading emerges when these ancient genetic mechanisms operate within uniquely human neural architecture. This explains why the SRD genes themselves do not show human-specific adaptations, yet reading remains a uniquely human skill.

These findings, when considered alongside the evolutionary conservation of the SRD genes and their association with HARs, paint a picture of reading as an emergent property arising from the complex interplay of ancient, conserved neural mechanisms and more recent regulatory innovations. This perspective helps explain both the universality of reading potential across human populations and the vulnerability of this skill to genetic and developmental perturbations.

### MECP2 and SRD Genes: A Shared Pathway in Reading Disorders

The overlap between MECP2-regulated pathways and the SRD genes provides a molecular framework for understanding why reading difficulties are common in Rett syndrome and potentially in other genetic syndromic disorders. Our findings suggest that *MECP2* may regulate the expression of multiple genes involved in the development of neural circuits critical for reading acquisition. Particularly relevant is our discovery that many SRD genes transition in their expression patterns around 24 pcw, corresponding to a crucial period when *MECP2* expression increases in the developing cortex (Feldman et al., 2016). This *MECP2* connection extends beyond Rett syndrome. Emerging evidence indicates that variation in MECP2 expression or function, even without clinical Rett syndrome, may impact reading-related cognitive processes (Gonzales & LaSalle, 2010).

The regulatory network involving MECP2 appears to influence multiple neurodevelopmental pathways shared across various disorders, including those affecting reading skill development. From a translational perspective, understanding how MECP2 interacts with SRD genes could inform therapeutic approaches not only for reading difficulties in Rett syndrome but potentially for broader nonspecific reading difficulties.

Recent therapeutic approaches targeting MECP2 pathways, including gene therapy and pharmacological modulation of downstream effectors, may eventually provide avenues for addressing specific reading-related deficits in both syndromic and nonsyndromic contexts. This observation exemplifies how the study of genetic syndromes with known etiologies, such as Rett syndrome, can illuminate the mechanisms underlying more common, genetically complex conditions, like specific reading (dis)ability (Palmieri et al., 2023). The MECP2 regulatory network may represent a convergent pathway through which diverse genetic variations can impact reading development, providing a molecular target for future precision interventions.

### Clinical Implications

Our findings have several clinical implications. The identification of two distinct functional gene clusters—one associated with early brain morphology development (before 24 pcw) and another with later synaptic signaling—suggests that SRD may manifest different neurobiological subtypes with potentially distinct clinical presentations. This finding aligns with previous clinical research, which has identified heterogeneous profiles in individuals with SRD (Pennington et al., 2012; Zoubrinetzky et al., 2014).

Our findings regarding the *MECP2* gene involvement provide a potential explanatory mechanism for why reading difficulties are observed in Rett and other syndromic disorders. The overlap between SRD gene networks and genes implicated in other neurodevelopmental conditions suggests that shared neurobiological pathways could inform assessment and intervention approaches across conditions. For example, the notable hub genes we identified (*NRXN1*, *CTNNA3*, *CTNNAP2*, *KIAA0319*, *CMIP*, *ROBO1*, *GRIN2B*) have been implicated in multiple neurodevelopmental disorders and may represent critical intervention targets.

The critical transition point around 24 pcw in gene expression patterns could inform developmental screening timelines and early intervention approaches. Disruptions during this key developmental window might contribute to later reading difficulties, suggesting the potential for earlier identification of children at genetic risk for SRD. This could enable preventative interventions before the reading instruction formally begins.

From a precision medicine perspective, our findings suggest that genetic profiling might eventually help identify which individuals with SRD would benefit most from specific intervention approaches. For instance, individuals with genetic variations affecting primarily the early-development gene cluster might benefit from interventions targeting foundational neural circuits, while those with variations in the later-expressed signaling genes might respond better to interventions focused on strengthening specific neural pathways involved in reading processes. However, judgments about the diagnostic utility of our results should be preceded by, and grounded in, careful work that connects different reading interventions to such pathways.

The evolutionary conservation of the SRD genes across species and their expression across multiple brain regions further support viewing SRD as part of a broader neurodevelopmental spectrum rather than an isolated condition. This perspective reinforces the value of comprehensive assessment approaches that consider potential comorbidities, as recommended by recent clinical guidelines (Snowling et al., 2020). Understanding the ancient evolutionary origins of the SRD genes also provides a biological explanation for why learning difficulties persist despite systemic efforts to remediate them in modern society.

### Limitations and Future Directions

Despite the comprehensive nature of our analysis, several limitations warrant consideration. First, our literature review, while extensive, may not have captured all relevant genes, particularly those published in non-English journals or identified through methodologies not included in our search criteria. Second, the evolutionary analyses were conducted using available reference genomes, which represent a small fraction of species diversity and may not capture the full evolutionary history of these genes. Furthermore, our transcriptomic analyses relied on publicly available data sets, which have inherent limitations in terms of sample size, developmental time points, and the brain regions represented. The Allen Brain Atlas, although extensive, primarily contains data from neurotypical individuals, which limits our ability to examine expression differences specifically in individuals with reading disabilities. Our single-cell RNA sequencing analysis was restricted to adult brain tissue, preventing direct examination of cell-type–specific expression during critical developmental periods for reading acquisition. The clustering approach we employed, while robust, necessarily simplified complex gene–gene relationships and may have obscured more nuanced interaction patterns relevant to reading development.

Finally, we acknowledge the gap between genetic findings and educational applications. While our results suggest potential neurobiological mechanisms underlying reading (dis)ability, translating these findings into effective interventions requires interdisciplinary efforts beyond the scope of this research. Future studies may focus on the functional validation of the SRD genes through animal models and human neuroimaging studies, as well as the examination of gene–environment interactions in reading development and longitudinal studies that track how genetic variants influence reading trajectories over time.

## Conclusions

Our findings challenge the notion of specialized genes for reading and, instead, position SRD as a disruption of evolved neurodevelopmental processes. The systemic nature of reading—where ancient genetic mechanisms interact with human-specific brain architecture—explains both its universality and vulnerability. Future systems-level analyses, integrating cross-species models and multi-omics data, will be essential to unravel how conserved molecular pathways enable uniquely human cognition. To comprehensively investigate SRD in particular and reading in general, it is imperative to evaluate system properties encompassing gene expression, methylation patterns, protein translation dynamics, and related factors. Such a holistic approach is indispensable for unraveling the intricate neurobiological underpinnings of SRD and advancing our understanding of its etiology. An investigation of the structural variation in the genome was a good starting point; we have learned a great deal, although we have not yet cracked the puzzle of the genetic bases of reading and reading difficulties.

## Author Contributions

**Pavel Dobrynin:** Conceptualization, Methodology, Investigation, Visualization, Supervision, Writing – original draft, Writing – review & editing. **Yi Zeng:** Investigation, Visualization. **Marina Norkina:** Investigation, Writing – review & editing. **Alina Fedorova:** Methodology, Investigation, Writing – review & editing. **Anna Zhuk:** Visualization, Writing – review & editing. **Elena L. Grigorenko:** Conceptualization, Methodology, Investigation, Funding acquisition, Supervision, Writing – original draft, Writing – review & editing.

## Data Availability Statement

All used data were collected from public repositories. IPython notebook with Python code used for analysis with PAML (Phylogenetic Analysis by Maximum Likelihood) and evolutionary novelty analysis is available in Supplemental Materials S10 and S11. We used the Google Colab environment to run Python. Any necessary intermediate files (i.e., gene alignments, filtered expression data) are available upon request to the corresponding author.

## Supplementary Material

10.1044/2025_JSLHR-25-00050SMS1 Supplemental Material S1 SRD Genes - Comprehensive catalog of 175 genes identified as putative candidates for Specific Reading Disability, including Ensembl ID, NCBI ID, and common gene name for reference and cross-database identification.

10.1044/2025_JSLHR-25-00050SMS2 Supplemental Material S2 Literature Review Methodology – Details on stepwise approach for systematic literature identification, including search criteria, filtering conditions, and resulting paper counts at each stage of the review process.

10.1044/2025_JSLHR-25-00050SMS3 Supplemental Material S3
Brain Regions in Transcriptome Analysis - Anatomical brain regions examined in the developmental transcriptome dataset with their corresponding acronyms and inclusion status for subsequent analysis.

10.1044/2025_JSLHR-25-00050SMS4 Supplemental Material S4 Differential Gene Expression Analysis - Complete output from Limma analysis identifying differentially expressed genes across developmental timepoints, with additional classification based on single-cell dataset clustering.

10.1044/2025_JSLHR-25-00050SMS5 Supplemental Material S5 Prenatal Gene Expression Enrichment - Gene ontology (GO) biological processes significantly enriched among genes upregulated during fetal development, including statistical metrics, pathway information, and gene identifiers.

10.1044/2025_JSLHR-25-00050SMS6 Supplemental Material S6 Postnatal Gene Expression Enrichment - Gene ontology (GO) biological processes significantly enriched among genes upregulated after birth, including statistical metrics, pathway information, and gene identifiers.

10.1044/2025_JSLHR-25-00050SMS7 Supplemental Material S7 Evolutionary Selection Analysis - Statistical summary of selection models (branch model, FEL, RELAX) applied to SRD genes, including test statistics and significance values for evolutionary constraint assessment.

10.1044/2025_JSLHR-25-00050SMS8 Supplemental Material S8 KEGG Pathway Enrichment - Results from gene set enrichment analysis against the KEGG database, highlighting significant biological pathways represented by SRD genes with statistical measures and gene identifiers.

10.1044/2025_JSLHR-25-00050SMS9 Supplemental Material S9 Reactome Pathway Analysis - Enrichment analysis results using the Reactome database, identifying significant functional pathways represented by SRD genes with corresponding statistical metrics.

10.1044/2025_JSLHR-25-00050SMS10 Supplemental Material S10 PAML and evolutionary novelty analysis.

10.1044/2025_JSLHR-25-00050SMS11 Supplemental Material S11 Raw data from HomoloGene, retrieved on 02/05/2023.

## Notes

**Publisher Note:** This article is part of the Forum: Research Symposium on Genetics in Communication Sciences and Disorders.
